# Regulatory Mechanisms of Estrogen on Vascular Ageing

**DOI:** 10.1155/2019/4859082

**Published:** 2019-04-28

**Authors:** Susana Novella, Ana P. Dantas, Carlos Hermenegildo, Ylva Hellsten

**Affiliations:** ^1^Department of Physiology, University of Valencia and INCLIVA Biomedical Research Institute, Valencia, Spain; ^2^Laboratory of Experimental Cardiology, August Pi i Sunyer Biomedical Research Institute (IDIBAPS), Hospital Clinic Cardiovascular Institute, Barcelona, Spain; ^3^Department of Nutrition, Exercise and Sports, University of Copenhagen, Denmark

Women can be considered hemodynamically younger than men of the same age, based on epidemiological studies establishing that the incidence of vascular diseases in women is relatively lower compared to that in aged-matched men. However, after menopause, these numbers increase to values that are close to those found in men. Vascular ageing is associated with structural and functional changes of the vascular wall, including endothelial dysfunction, arterial stiffening, and remodelling, as well as impaired angiogenesis, which become major risk factors in the development of cardiovascular disease. Data from clinical and basic research have established that vascular ageing does not follow the same timeline in women as it does in men. Although vascular dysfunction and cardiovascular risk factors are comparable in elderly men and women, the rate of change is lower in women during their fertile years. This age- and sex-related difference in the progression of vascular dysfunction is not completely understood, although experimental and observational clinical studies have attributed most of the vascular protection in women to estrogen and its modulatory action in the vascular system via interaction with its receptors ER*α*, ER*β*, and G protein-coupled estrogen receptor (GPER).

Nevertheless, there is much controversy surrounding the clinical use of estrogen as a therapeutic strategy in primary and secondary prevention of vascular disease in women. Contrary to the observational studies suggesting the value of estrogen to vascular health in women, clinical trials such as the Women's Health Initiative (WHI) reported an increase in cardiovascular events in women treated with estrogen replacement therapy compared with those treated with placebo. A main reason for the discrepancy among studies is that women enrolled in the clinical trials were on average 10 years older than those included in the observational studies. When analyses are done by the age group, it is revealed that estrogen therapy produces more favourable results in younger women (closer to onset of menopause) than in the late average onset enrolled in most trials. These analyses led scientists to conceive the so-called “timing hypothesis,” which relies on the concept that estrogen has beneficial effects only if taken before, or close to, the onset of menopause, at which time the detrimental effects of ageing have not yet been established in the vasculature.

Among the mechanisms underlying the estrogen-mediated effects on vascular function are anti-inflammatory and antioxidative actions, as well as epigenetic modifications, all of which are influenced by ageing. In fact, a great burden of oxidative stress is seen during ageing and menopause which can contribute to vascular dysfunction. That way, oxidative stress together with the inflammatory activation of the vascular endothelium induces a wide range of local and systemic responses, such as the expression of adhesion molecules, the production of chemotactic factors, and free radicals that could regulate the vascular effects of estrogen. Thus, the vasoprotective effects seen in younger women, in the perimenopause or close to menopause onset, could become detrimental when women get older.

This special issue presents novel and updated research regarding the mechanisms by which estrogen modulates metabolism and vascular homeostasis in different stages of women's health including menarche, pregnancy, and menopause. With special focus on oxidative stress, these mechanisms could be pivotal for the development of novel therapeutic strategies to prevent and treat vascular disease in women. The manuscripts within this special issue are all equally recommended by the editors, and they contain especially interesting points worth commenting on.

Estrogens are important modulators of lipid metabolism and contribute to the low prevalence of atherosclerotic vascular disease. However, estrogen promotes the synthesis of triglycerides (TG) in the liver and secretes TG into the circulation as very-low-density lipoprotein (VLDL) particles, being the hypertriglyceridemia one of the five pathological traits that characterize metabolic syndrome. In fact, an association exists between early menarche and an increased risk of metabolic syndrome. To better understand this relation, H.-S. Lee et al. identified through a GWAS-based pathway analysis an association among type 2 diabetes mellitus, stress-activated protein kinase (SAPK) signalling, and Jun amino-terminal kinase (JNK) cascade pathways with TG including the genetic interaction with age at menarche.

Type 2 diabetes is an age-associated disease in which oxidative stress is involved, the prevalence is higher in men than in women, and therefore, there are sex-associated differences. In the original study developed by A. Díaz et al., the role of estrogens in protecting against oxidative stress in type 2 diabetic Goto-Kakizaki male and female rats is evaluated. Performing an in-depth study in glucose metabolism, by positron emission tomography, metabolomics, and mitochondrial activity measurements, these authors provide evidence for the beneficial effects of estrogen in ovariectomized type 2 diabetes female rats.

Estrogen also plays a pivotal role in the control of vascular function during pregnancy. Normal gestation is associated with a mild oxidative stress, which is increased in pregnancy complications, such as preeclampsia and intrauterine growth restriction. Estrogen regulates uterine artery function via upregulation of endothelial nitric oxide synthase (eNOS) in endothelial cells and the large-conductance Ca^2+^-activated K^+^ (BKCa) channel in vascular smooth muscle cell. The review presented by X.-Q. Hu et al. provides an updated understanding of the regulation of the estrogen-NOS-NO-KCa pathway by reactive oxygen species in uteroplacental tissues and gives an overview of a link between oxidative stress and uteroplacental dysfunction in pregnancy complications.

Female sex hormones also play a predominant role in varicose physiopathology, especially considering the influence of sex and pregnancy on varicose vein development. The original study proposed by N. García-Honduvilla et al. is aimed at verifying the involvement of steroid receptors and progesterone, estrogen, and androgen receptors in varicose vein development, focusing on sex differences. In both sexes, they observed an increase in sex hormone receptors under varicose vein conditions and highlight the importance of the redistribution of these receptors.

Finally, estrogen also modifies cardiovascular function by modulating gene expression, either directly acting as transcriptional factors or indirectly via epigenetic regulation. In this sense, miRNAs have emerged as a new regulatory mechanism of both physiological and pathological processes, as they regulate gene expression profiles at the posttranscriptional level. Epigenetic changes in the vascular action of estrogens in ageing are deeply reviewed in the work by D. Pérez-Cremades et al. Emerging studies propose a role for miRNA in the vascular effects mediated by estrogens, and in this work, the authors highlight the current knowledge on the role of estrogen-sensitive miRNAs and their influence in regulating vascular ageing. This review sheds light on the potential of translating miRNA research into the clinic, using miRNAs as potential tools for the diagnoses and/or prognoses of cardiovascular disease, as affordable and noninvasive biomarkers, and as a therapeutic tool for regulating (silencing or increasing) miRNA levels.

